# Analysis of HLA-G 14 bp Insertion/Deletion Polymorphism and HLA-G, ILT2 and ILT4 Expression in Head and Neck Squamous Cell Carcinoma Patients

**DOI:** 10.3390/diseases12020034

**Published:** 2024-02-08

**Authors:** Vladimira Durmanova, Miroslav Tedla, Dusan Rada, Helena Bandzuchova, Daniel Kuba, Magda Suchankova, Agata Ocenasova, Maria Bucova

**Affiliations:** 1 Institute of Immunology, Faculty of Medicine, Comenius University in Bratislava, 811 08 Bratislava, Slovakia; magda.suchankova@fmed.uniba.sk (M.S.); agata.ocenasova@fmed.uniba.sk (A.O.); maria.bucova@fmed.uniba.sk (M.B.); 2 Department of Ears, Nose and Throat and Head and Neck Surgery, Faculty of Medicine, University Hospital Bratislava, Comenius University in Bratislava, 851 07 Bratislava, Slovakia; miro.tedla@gmail.com (M.T.); dusan.rada@centrum.cz (D.R.); 3 National Transplant Organisation, 831 01 Bratislava, Slovakia; helena.bandzuchova@nto.sk (H.B.); daniel.kuba@nto.sk (D.K.)

**Keywords:** HLA-G, ILT receptor, head and neck squamous cell carcinoma, mRNA expression, real-time PCR

## Abstract

HLA-G is the checkpoint molecule involved in the suppression of the immune response. Increased expression of HLA-G and its ILTs receptors have been correlated with tumor progression in various cancer types. In head and neck squamous cell carcinoma (HNSCC) tumors, the effect of HLA-G, ILT2 and ILT4 expression on cancer development has to be explained. The 34 HNSCC patients and 98 controls were genotyped for the HLA-G 14 bp ins/del polymorphism. In HNSCC lesions, HLA-G, ILT2 and ILT4 mRNA expression was analysed using real-time PCR. The association between HLA-G, ILT2 and ILT4 mRNA expression and clinical variables (age at onset, TNM staging system and p16 positivity) was also evaluated. No genetic association between the HLA-G 14 bp ins/del and HNSCC risk was detected (*p* > 0.05). However, in the non-metastatic HNSCC group, a significantly higher HLA-G mRNA expression was noted in tumors in the T4 stage compared to those in the T1 and T2 stages (*p* = 0.0289). ILT2 mRNA expression was significantly increased in non-metastatic vs. metastatic tumors (*p* = 0.0269). Furthermore, a significantly higher ILT4 mRNA expression was noted in tumors in the T1+T2 stage compared to those in the T3 stage (*p* = 0.0495). Our results suggest that the HLA-G molecule creates an immunological microenvironment involved in HNSCC development.

## 1. Introduction

Head and neck squamous cell carcinoma (HNSCC) is the sixth most common cancer worldwide, with an incidence of approximately 890,000 cases and around 450,000 deaths per year [1]. It usually originates from the mucosal epithelium of the oral cavity, hypopharynx, oropharynx and larynx. Long-term exposure to tobacco, alcohol and human papilloma virus (HPV) infections are recognized as the established risk factors for HNSCC. In general, the risk of developing HNSCC is two to four times higher in men compared to women. The classification of HNSCC is determined by the tumor–node–metastasis (TNM) staging system, with additional information relevant to HPV positivity [2].

Suitable biomarkers that could predict the risk of HNSCC are still a subject of investigation. One of the molecules that may contribute to the development of cancer is the human leukocyte antigen G (HLA-G). HLA-G belongs to the non-classical HLA I molecules and is linked with the suppression of immune reactions. During pregnancy, this molecule is expressed in the extravillous cytotrophoblast, which plays a crucial role in protecting the fetus from the mother’s immune response [3]. Additionally, HLA-G as an immunomodulatory molecule has been associated with many diseases, including cancer [4]. The overexpression of HLA-G has been observed in a variety of cancers, including lung, breast, ovarian, renal, gastric and colorectal cancer as well as hematological malignancies [5]. In cancer, HLA-G is involved in the progression of primary tumors to the metastatic stage, followed by poor prognosis and overall survival [6].

Through the alternative splicing of the primary transcript, four membrane-bound isoforms HLA-G1, -G2, -G3 and -G4 and three soluble isoforms HLA-G5, -G6 and -G7 are generated [7]. HLA-G isoforms mediate an inhibition of the cytotoxic activity of natural killer (NK) cells and CD8+ T cells, the alloproliferative response of CD4+ T cells, the inhibition of dendritic cell maturation and the differentiation of CD4+ T-cells into regulatory T cells [8]. Via trogocytosis, HLA-G can stimulate the production of immunosuppressive CD4+HLA-G+ and CD8+HLA-G+ T cells and HLA-G+ APCs, such as DC-10 and HLA-G+ macrophages [9]. Moreover, sHLA-G binds CD8 and activates the apoptosis of CD8-bearing cells via Fas/Fas ligand interaction [10].

The HLA-G molecule’s immunosuppressive response is primarily mediated by its interaction with two important inhibitory receptors present on immune cells, namely, immunoglobulin-like transcript 2 (ILT2/CD85j/LILRB1) and immunoglobulin-like transcript 4 (ILT4/CD85d/LILRB2). ILT2 is present on T and B cells, natural killer (NK) cells, myeloid-derived suppressive cells (MDSCs), dendritic cells (DCs) and monocytes/macrophages and mainly recognizes HLA-G1 and -G5 isoforms associated with β2M. The ILT4 receptor is mainly expressed on neutrophils, monocytes/macrophages, basophils, DCs and MDSCs and binds the HLA-G2 and HLA-G6 isoforms and also β2M-free HLA-G1 and HLA-G5 [11]. The binding of HLA-G to ILTs activates the phosphorylation of their ITIM domains, which are thought to recruit the protein tyrosine phosphatase SHP-1, leading to a suppression of the signaling cascade. ILT2/4s are more accessible to bind to the HLA-G dimer than the HLA-G monomer, resulting in much stronger inhibitory signals [12]. In addition to immune cells, tumor cells also show abundant ILT2 and ILT4 expression. The overexpression of ILT2 and ILT4 receptors was found in various cancers and was related to de novo expressions of HLA-G [13,14,15,16,17,18,19]. Upregulated ILT2 and ILT4 expression was associated with shorter overall survival, lymph node metastasis and a smaller number of tumor-infiltrating lymphoid cells [16,19,20,21,22,23,24].

It is known that the HLA-G expression may be affected by the genetic variants in the 5′URR (upstream regulatory region) and 3′UTR (untranslated) regions. The HLA-G 14 bp ins/del polymorphism (rs16375), located in the 3′UTR, accounts for the most studied polymorphism among different diseases. The 14 bp deletion (del) allele was found to increase the protein expression level, whereas the 14 bp insertion (ins) allele was associated with decreased HLA-G expression [25]. Many studies assessed the association of the HLA-G 14 bp ins/del variant with different types of cancer risks, but with contradictory results [26]. The latest meta-analyses of Jiang et al. [27] revealed a protective role of the HLA-G 14 bp ins/ins genotype compared to the HLA-G 14 bp del/del genotype (OR = 0.65, *p* < 0.05) in breast cancer patients. In oesophageal cancer, they also reported a protective effect of a 14 bp ins/ins in a dominant genetic model (OR = 0.66, *p* < 0.05). The impact of HLA-G expression on clinical variables in HSNCC has been analysed in few studies so far. The expression of HLA-G was found to be significantly higher in tumor lesions from patients with head and neck squamous cell carcinoma (HNSCC) and oral squamous cell carcinoma (OSCC) compared to controls. Furthermore, HLA-G expression was positively correlated with the histological grade [28], metastasis [28,29,30,31,32,33] and HPV presence [34,35]. The overexpression of HLA-G negatively correlated with overall survival in OSCC patients [30,33].

No study evaluated the association between the HLA-G 14 bp ins/del polymorphism, HLA-G, ILT2 and ILT4 expression and the risk of HNSCC until now. Thus, the aim of this study was to analyse the correlation between an HLA-G 14 bp ins/del polymorphism, HLA-G, ILT2 and ILT4 expression and clinical variables in HNSCC patients.

## 2. Materials and Methods

### 2.1. Study Subjects

We investigated 34 patients (33 males and 1 female) suffering from head and neck squamous cell carcinomas (HNSCC) localised in oropharynx, hypopharynx, larynx and oral cavity. The average age of patients diagnosed with HNSCC was 62.32 ± 6.63 years. Patients were randomly recruited from Clinics of Otorhinolaryngology and Head and Neck Surgery, Faculty of Medicine, Comenius University and University Hospital Bratislava, Slovakia. Two histopathologists approved the diagnosis according to the latest WHO classification criteria [36]. Patients’ clinical data include age at onset, diseased site, TNM staging, tobacco status and p16 positivity. Blood samples and tumor excisions were obtained from the patients on the day of surgical treatment. The control group comprised 98 age-matched unrelated individuals (92 males and 6 females) with a mean age of 63.10 ± 7.41 years.

All study participants provided written informed consent to enroll in the study and for personal data management. The Ethics Committee of the Faculty of Medicine, Comenius University and University Hospital in Bratislava has approved the study. All the investigations were carried out in accordance with the International Ethical Guidelines and the Declaration of Helsinki.

### 2.2. PCR Analysis of HLA-G 14 bp ins/del Polymorphism

A modified salting-out procedure was used to isolate genomic DNA from 2 mL of EDTA-treated peripheral HNSCC and control blood samples [37]. HLA-G 14 bp ins/del polymorphism (rs66554220) at the 3′UTR region was analysed via PCR using forward primer 5′GTGATGGGCTGTTTAAAGTGTCACC-3′ and reverse primer 5′GGAAGGAATGCAGTTCAGCATGA-3′, as described by Hviid et al. [38]. The PCR reaction mixture, with a total volume of 25 μL, consisted of 50 ng of genomic DNA, 0.2 mM of each dNTP (Thermo Fisher Scientific, Waltham, MA, USA), 1.5 mmol MgCl2 (Thermo Fisher Scientific, USA), 1 U of Taq DNA polymerase (Thermo Fisher Scientific, USA) and 10 pmol of forward and reverse primer. The PCR protocol involved denaturation at 95 °C for 3 min, followed by 30 cycles of denaturation at 95 °C for 1 min, annealing at 64 °C for 1 min and elongation at 72 °C for 1 min each. The final elongation step was performed at 72 °C for 10 min. The PCR products were separated in 3.0% agarose gel for 30 min and then examined under UV light. Using a 100 bp DNA ladder (Solis BioDyne, Tartu, Estonia), PCR fragments of 224 bp (14 bp insertion) and 210 bp (14 bp deletion) were detected.

### 2.3. Real-Time RT-PCR Analysis of HLA-G, ILT2 and ILT4 Expression

Tissue samples (3 × 3 mm) excised from different tumor sites of pharynx and larynx were collected in RNA later solution (RNA laterTM, Qiagen, Hilden, Germany). The tissue was disrupted in TRIzol lysis buffer (Life Technologies, Carlsbad, CA, USA) by means of TissueLyser II system (Qiagen, Germany). Total RNA was extracted using the manufacturer’s protocol (TRIzol RNA extraction protocol, Life Technologies, USA). The RNA pellet was dissolved in RNase free water and stored at −70 °C. TRIzol lysis buffer was used to isolate total RNA from cell lines and PBMC (Life Technologies, USA).

High-Capacity RNA-to-cDNA Kit was used to synthesize cDNA from total RNA (Applied Biosystems, Waltham, MA, USA). RNA mixture containing RNA at a concentration of 1 µg was incubated at 25 °C for 10 min, followed by incubation at 42 °C for 60 min, with a total volume of 20 µL. The reaction was completed via heating at 95 °C for 5 min. cDNA was used as template for HLA-G amplification. The HLA-G specific primers and probes were chosen to amplify all isoforms of HLA-G transcripts (HLA-G/F: 5′-CTGGTTGTCCTTGCAGCTGTAG-3′, HLA-G/R: 5′-CCTTTTCAATCTGAGCTCTTCTTTCT-3′, HLA-G probe: 5′-CACTGGAGCTGCGGTCGCTGCT-3′) [39]. TaqMan probes were labelled with 6-carboxyfluorescein at the 5′ end (FAM reporter) and 6-carboxytetramethylrhodamine at the 3′ end (TAMRA quencher) and recognised a sequence located between PCR primers. A 25 μL reaction mixture for real-time PCR contained 1x TaqMan^®^ Gene Expression Master Mix (Thermo Fischer Scientific, USA), 200 nM of specific primers, 150 nM of specific TaqMan probe and 3 μL of cDNA. cDNA of ILT2 and ILT4 was amplified using TaqMan gene expression assays (ILT2 Hs00366806_m1, ILT4 Hs00275975_ml, Thermo Fischer Scientific, USA) according to the manufacturer’s instructions. TaqMan probes FAM-MGP were designed to span an exon junction and were validated by manufacturer (Applied Biosystems, USA). The cDNA was amplified using the ABI Prism7000 Sequence Detection System in accordance with the manufacturers recommendations (Applied Biosystems, USA). The PCR protocol involved denaturation at 95 °C for 10 min, followed by 40 cycles of denaturation at 95 °C for 15 s and annealing at 60 °C for 1 min. Glyceraldehyde-3-phosphate dehydrogenase (GAPDH) gene was expressed using specific probe and primers and was used as endogenous control (GAPDH/F: 5′-CATGGGTGTGAACCATGAGAA-3′, GAPDH/R: 5′-GGTCATGAGTCCTTCCACGAT-3′, GAPDH probe: 5′-AACAGCCTCAAGATCATCAGCAATGCCT-3′). Values of cycle threshold (CT) were determined via automated threshold analysis using ABI Prism software (Version 3.7). To calculate the changes in gene expression of individual tissue samples, comparative cycle threshold method (2^−ΔΔCT^) was used. The HLA-G expression in tissue samples was computed to the expression in control PBMC cells (expression = 1) to obtain relative expression value.

The following human cell lines were used for a specificity check of primers: leukemia cell lines lacking HLA-G (0.221, K562, kindly provided by Cancer Research Institute BMC, SAS, Bratislava), HLA-G1 transfected cell lines (0.221-G1, K562-G1), HLA-G2 transfected cell line (K562-G2, both kindly provided by Dr. E. Weiss from Munich, Germany) and 0.221 cell line treated with DNA hypomethylation agent 5-aza-2‘-deoxycytidine (causing conversion to HLA-G positive counterpart). JEG-3 (ATCC: HTB-36) and JAR (ATCC: HTB-144) choriocarcinoma cell lines were used as positive and negative controls for HLA-G, respectively.

### 2.4. RT-PCR of HLA-G Isoforms

Specific HLA-G isoforms (HLA-G1, -G2, -G5 and -G6) were evaluated via reverse transcriptase (RT)-PCR analysis using primers described in the study of Yao et al. [40]. cDNA, prepared elsewhere, was subjected to amplification using PCR conditions as follows: incubation of DNA polymerase at 95 °C for 15 min, followed by cDNA denaturation at 95 °C for 15 s, annealing at 62 °C for 15 s and elongation at 72 °C for 60 s. The final extension lasted for 7 min at 72 °C. The number of PCR cycles was determined experimentally: 35–40 cycles for HLA-G isoforms and 25 cycles for GAPDH amplification as endogenous control. PCR products were separated in 2% agarose gel stained with GelRed (Biotium, Fremont, CA, USA) and visualized under UV light. A 100 bp DNA ladder (Solis BioDyne, EU) was used to analyse PCR product size.

### 2.5. Statistical Analysis

HLA-G 14 bp ins/del allele and genotype distribution were determined via direct counting. Genotypes were checked for their departure from Hardy–Weinberg equilibrium (HWE) using the chi squared goodness-of-fit test. The Pearson chi-squared statistical test was used to evaluate the differences in HLA-G 14 bp ins/del allele and genotype frequencies between the two groups studied (HNSCC patients vs. control group) (GraphPad Software, Inc., San Diego, CA, USA). The *p* values and odds ratios (OR) with 95% confidence intervals (95% CI) were subjected for analysis in codominant, dominant and recessive inheritance models. The SNPstats web software available at https://www.snpstats.net/start.htm (accessed on 15 February 2023) was used to perform multivariate logistic regression analysis, adjusting for age and sex as possible influencing factors. Student’s *t*-test with Welch correction was used to investigate the correlation between relative HLA-G, ILT2 and ILT4 expression and clinical variables such as age at onset, TNM staging and p16 positivity. Finally, Pearson correlation was used to analyze the differences in relative mRNA expression of HLA-G, ILT2 and ILT4. A *p* value of less than 0.05 was considered statistically significant.

## 3. Results

### 3.1. Characteristics of the Study Groups

Table 1 displays the demographic and clinical features of the study. The research enrolled 34 head and neck squamous cell carcinoma (HNSCC) patients and 98 control individuals. The difference between the HNSCC group and controls in relation to gender was not statistically significant (*p* = 0.79). Men had a higher prevalence in both HNSCC patients (97.06%) and controls (93.88%). No statistically significant difference between the studied groups was found in relation to age at examination (*p* = 0.57). The mean age at onset of HNSCC patients was 61.65 ± 6.18 years. Out of 34 HNSCC patients, 31 patients had a primary diagnosis and 3 had a relapse. Tumors were localised in the oropharynx (n = 6), hypopharynx (n = 8), larynx (n = 14), oral cavity (n = 1) and lymph nodes in the neck (n = 5). The majority of HNSCC patients had a positive tobacco status (30 vs. 4).

In relation to the TNM staging system, 1 patient had a tumor in the T1 stage, 6 had tumors in the T2 stage, 6 had tumors in the T3 stage, 16 had tumors in the T4 stage and 5 had tumors that were not classified. Regarding lymph node involvement, 13 patients had tumors in the N0 stage, 6 had tumors in the N1 stage, 10 had tumors in the N2 stage and 5 had tumors in the N3 stage. Distal metastasis was found in six patients. The p16 marker associated with HPV presence was observed in six patients.

### 3.2. Analysis of HLA-G 14 bp ins/del Polymorphism and HLA-G mRNA Expression in HNSCC Patients and Control Group

Table 2 summarises the HLA-G 14 bp ins/del allele and genotype frequencies among HNSCC patients and controls. The genotype distribution fit the HWE in HNSCC patients (χ^2^ = 3.03, *p* = 0.08) as well as in controls (χ^2^ = 0.17, *p* = 0.68). No statistically significant differences in the HLA-G 14 bp ins/del allele (*p* = 0.65, OR = 1.19) and genotype (*p* > 0.05, OR = 0.82–1.95) frequencies were determined between HNSCC patients and the control group. However, in the HNSCC group, there was a higher OR of 1.95 in the 14 bp ins/del genotype carriers in the codominant model and a higher OR of 1.78 in the dominant model compared to other genotype carriers. Multivariate analysis of association between the 14 bp ins/del polymorphism and HNSCC risk adjusted for age and sex revealed no changes in comparison with the univariate analysis (*p* > 0.05, OR = 0.76–1.87, Table 2).

Correlation analysis of HLA-G 14 bp ins/del polymorphism with relative HLA-G mRNA expression was performed in HNSCC patients. No statistically significant difference was found between individual HLA-G 14 bp ins/del genotypes and relative HLA-G mRNA expression, as shown in Table 3.

### 3.3. Association of HLA-G Isoforms with HLA-G mRNA Expression in HNSCC Patients

In the HNSCC group, we investigated the presence of the membrane-bound isoforms HLA-G1 and HLA-G2 and the soluble isoforms HLA-G5 and HLA-G6. The most frequent were carriers of the single HLA-G1 isoform (26.47%), followed by carriers of the HLA-G1, HLA-G2 and HLA-G5 isoforms (17.65%). The carriers of the HLA-G1 and HLA-G2 isoforms accounted for 14.71%, followed by carriers of the HLA-G1, HLA-G2 and HLA-G6 isoforms (11.76%). Other combinations of the above-mentioned isoforms account for 29.41%. No association of HLA-G isoforms with HLA-G mRNA expression in HNSCC patients was determined (*p* > 0.05.

### 3.4. Association of HLA-G, ILT2 and ILT4 mRNA Expression with Clinical Variables in HNSCC Patients

An analysis of association between HLA-G, ILT2 and ILT4 mRNA expression and the clinical variables age at onset, tumor staging, node involvement, distal metastasis and p16 positivity was performed in HNSCC patients. There was no statistically significant association between relative HLA-G mRNA expression and the abovementioned clinical variables in the whole HNSCC group (Table 4).

However, in the non-metastatic HNSCC group, a significantly higher relative HLA-G mRNA expression was found in patients with tumors in the T4 stage as compared to those with tumors in the T1 and T2 stages (1.480 ± 1.801 vs. 0.2680 ± 0.3137, *p* = 0.0289, Table 5).

The analysis of association between ILT2 mRNA expression and clinical variables in HNSCC patients is shown in Table 6. There was no statistically significant association between relative ILT2 mRNA expression and clinical variables such as age at onset, tumor staging, node involvement and p16 positivity. However, in the tumors of HNSCC patients with no distal metastasis, there was a significantly higher relative ILT2 mRNA expression compared to those of patients with metastasis (0.2107 ± 0.3135 vs. 0.05533 ± 0.07611, *p* = 0.0269). Concerning ILT4 mRNA expression, in HNSCC patients with tumors in the T1+T2 stage, there was a significantly higher relative ILT4 mRNA expression compared to those with tumors in the T3 stage (0.1880 ± 0.1259 vs. 0.0693 ± 0.03798, *p* = 0.0495, Table 6).

### 3.5. Association of HLA-G with ILT2 and ILT4 mRNA Expression in HNSCC Patients

The Pearson correlation between HLA-G and ILT2 and ILT4 mRNA expression was also calculated. In the HNSCC group, there was a significantly higher relative ILT2 mRNA expression compared to ILT4 mRNA expression (0.183 ± 0.2913 vs. 0.108 ± 0.1108, r = 0.7755, *p* < 0.0001).

## 4. Discussion

HLA-G is an immune checkpoint molecule involved in the suppression of both innate and adaptive immune responses [41]. Aberrant HLA-G expression in cancers was first reported in 1998 in melanoma cells [42], and since then, HLA-G has been evaluated worldwide in malignant samples of various cancer types [43]. Studies have found that increased expression of HLA-G in cancers is associated with advanced tumor stage, metastasis status and poor disease outcomes [6]. Thus, HLA-G may serve as a new biomarker of cancer development.

Several studies reported upregulated HLA-G expression in HNSCC patients using IHC staining, quantitative RT-PCR or the ELISA method compared to the control groups [28,31,32,33,44]. The difference in HLA-G expression between benign and metastatic HNSCC and OSCC tumors was also described. Fregonezi et al. [45] reported the overexpression of HLA-G in benign oral lesions and low expression in premalignant and malignant OSCC lesions. In contrast, Gonçalves et al. [30] found that HLA-G expression was significantly higher in metastatic OSCC than in nonmetastatic OSCC. Additionally, OSCC patients with a higher expression of HLA-G exhibited a tendency toward shorter survival (16 months) compared to those with lower expression of HLA-G (22 months). The enhanced HLA-G expression in lip carcinogenesis [46], oral precancerous lesions [32] and intraoral mucoepidermoid carcinoma [47] has been also identified. As gene polymorphisms can have an impact on the expression level of HLA-G, the association between the HLA-G 14 bp ins/del and HLA-G mRNA expression with HNSCC risk was studied. It has been shown that the HLA-G 14 bp insertion variant in the 3′ UTR is linked with decreased HLA-G expression [25]. The HLA-G 14 bp variant has been related to various diseases, including autoimmune diseases, infectious diseases and cancer [26,48].

In our study, we evaluated the HLA-G 14 bp ins/del allele and genotype frequencies of 34 HNSCC patients and compared them with 94 controls. No association between the HLA-G 14 bp ins/del polymorphism and HNSCC risk was detected. Furthermore, no correlation between the 14 bp ins/del and HLA-G mRNA expression was found. Agnihotri et al. [49] revealed that the tumors of carriers of the 14 bp ins/del + ins/ins genotypes and ins allele were significantly more pronounced in HNSCC patients compared to controls. In contrast, Wang et al. [50] reported the association of the 14 bp ins/ins genotype with a decreased OSCC risk in both the codominant model (*p* = 0.044) and the log-additive model (*p* = 0.044).

Increased HLA-G expression has also been associated with a risk of HPV infection, which is one of the causative agents for head and neck cancer [34,35]. Association analyses of the HLA-G 14 bp variant with HPV risk have yielded inconclusive results. According to a meta-analysis of 593 cases and 702 controls, there was no significant association between the HLA-G 14 bp variant and the risk of HPV infection [26]. This is in agreement with our results, which also revealed no correlation between the HLA-G 14 bp polymorphism and HPV positivity (data not shown).

Regarding the expression of HLA-G isoforms in the HNSCC group, our results revealed the predominance of carriers with the HLA-G1 isoform, followed by carriers of the HLA-G1, HLA-G2 and HLA-G5 isoforms. The third most frequent were carriers of the HLA-G1 and HLA-G2 isoforms. Earlier studies have reported an association between the HLA-G1 and HLA-G5 isoforms and primary ESCC [51] and also between HLA-G7 and HNSCC, particularly in HPV positive tumors [35].

Cancer therapy may also have an impact on the expression of HLA-G. It was found that radiotherapy decreased HLA-G expression on tumor cells as well as on tumor-infiltrating mononuclear cells [52]. The impact of the HLA-G 14 bp variant on the chemotherapy response has also been studied. It was shown that carriers of the HLA-G 14 bp ins allele lacked a complete first-line chemotherapy response in metastatic colorectal cancer patients [53]. In our study, the HLA-G expression was not affected by cancer treatment, as we used samples from patients before the initiation of systemic therapy.

In our study, an association between HLA-G, ILT2 and ILT4 mRNA expression and clinical variables such as age at onset, TNM staging system and p16 positivity, which is connected with HPV infection, was evaluated. In the non-metastatic HNSCC group, a significantly higher HLA-G expression was noted in tumors in the T4 stage compared to those in the T1 and T2 stages. However, no association between HLA-G mRNA expression and age at onset, lymph node status, distal metastasis and p16 positivity was found. Our findings are in partial agreement with the studies of Wang et al. [50] and Imani et al. [33], which found that serum sHLA-G levels and the HLA-G staining intensity of squamous cell carcinoma cells increased with increasing TNM stage classification of the tumors of OSCC patients (*p* < 0.05). Shen et al. [28] reported a positive association between HLA-G mRNA expression and histological grade; however, no association was found in relation to gender, TNM stage and lymph nodal metastasis in HNSCC patients. One study found no association between HLA-G expression and clinical parameters such as tumor size, lymph node status, distant metastasis and clinical stage in oral tongue squamous cell carcinomas (SCCs) and lower lip SCCs [54]. Other studies noted an enhanced expression of HLA-G mRNA in HPV-positive HNSCC tumors [34,35].

It was found that SINE-VNTR-Alu (SVA) retrotransposon insertion polymorphisms localized in the MHC genomic region can modify mRNA expression both within (cis) and outside (trans) the region. The retrotransposon insertion can create binding sites for transcription factors or act as sources of regulatory noncoding RNAs that affect mRNA transcription. In relation to the HLA-G-207 transcript, it was observed that R_SVA_24 insertion homozygotes are associated with downregulation of gene transcription. Moreover, this transcript has not be translated into a functional protein [55]. The absence of a 14 bp insertion in the HLA-G 3′UTR region is associated with high HLA-G production, which can be affected by the presence of retrotransposon insertion polymorphisms that have an impact on the production of stable mRNA and/or the affinity for miRNA.

The interaction between HLA-G and ILTs receptors is regarded as an immune checkpoint; thus, we have analyzed the relationship between HLA-G and the expression of ILT2 and ILT4 in HNSCC samples. Upon HLA-G binding, ILT2 and ILT4 receptors initiate a cascade of inhibitory signals, leading to the suppression of immune cells [11]. The expression of ILT2 and ILT4 in immune cells of various lineages has been enhanced via the activity of HLA-G [56]. Tumor cells, in addition to immune cells, have also shown abundant ILT2 and ILT4 expression. The overexpression of ILT2 was found in breast cancer, gastric cancer, colorectal cancer, T cell lymphomas and chronic lymphocytic leukemia [13,14,17,19,57]. ILT4 was reported to be expressed in non-small cell lung cancer (NSCLC), colorectal cancer and breast cancer [15,16,18,19,20]. Upregulated ILT2 and ILT4 expression has been associated with lymph node metastasis, shorter overall survival and a lower number of tumor-infiltrating lymphoid cells [15,16,19,20,21,22,23,24].

In our study, a significantly increased ILT2 mRNA expression was detected in non-metastatic compared to metastatic patients. Furthermore, a significantly higher ILT4 expression was noted in tumors in the T1+T2 stage compared to those in the T3 stage. Increased mRNA expression of ILT2 and ILT4 in early tumor development should be explained by the heterogeneity in HLA-G expression and the number of immune cells within tumor tissue. Inter-patient, inter-tumor and intra-tumor heterogeneity in HLA-G expression, as well as tumor-infiltrating immune cells in various types of malignancies, has been described previously [58,59]. A study by Rouas-Freiss et al. [60] demonstrated the heterogeneity in HLA-G, ILT2 and ILT4 expression in tumor cells and in tumor-infiltrating CD4+ILT2+ and CD8+ILT2+ T cells within renal tumor areas. Increased production of ILT2 and ILT4 in our study might also be explained by the induction of immune tolerance in the early stages of HNSCC development. The expression of the ILT2 receptor was found in gastric cancer biopsies with various differentiation degrees and pathologic types, and its expression has correlated with disease stage and tumor size [57]. The ILT4 receptor was reported to be overexpressed in earlier tumor stages (pT1-T2) in patients with oesophageal adenocarcinoma [61]. Higher ILT4 expression levels were significantly associated with a shorter survival of gastric and colorectal cancer (CRC) patients with an early, as well as an advanced, disease stage [19,24].

In our study, we also found a significantly higher ILT2 mRNA expression compared to ILT4 mRNA expression in the HNSCC group. One explanation is the differential presence of ILTs receptors on immune cells. ILT2 is mostly expressed by lymphoid and myeloid cells, whereas the expression of ILT4 is myeloid-specific [11]. Furthermore, it was found that the expression of ILTs receptors should be induced by various factors, such as inflammatory cytokines (IL-10, TGF-β, IFN-α, IL-16 or thrombopoietin), interactions with regulatory T cells, immunosuppressive drugs (dexamethasone, niflumic acid, rapamycin and resveratrol) and Toll-like receptor (TLR) signalling [62].

A knowledge of the enhanced expression of HLA-G and ILTs in HNSCC progression should be used in cancer immunotherapy. In order to develop a novel approach for cancer treatment, it is crucial to incorporate HLA-G antibodies together with antibodies that target immune checkpoints, such as PD-1, CTLA-4 and TIM-3 [63]. Nowadays, the HLA-G/ILT2 pathway is considered a new target for immune checkpoint therapy. A study by Dumont et al. [64] characterized the CD8+ILT2+ lymphocyte population as more cytotoxic compared to CD8+PD1+ cells, which are the main target of immune checkpoint therapy. The CD8+ILT2+ cells are suppressed specifically by HLA-G. It has been shown that the usage of HLA-G antibodies has restored CD8+ILT2+ cell activity and has blocked the immunosuppressive effect of HLA-G. Similarly, the usage of anti-ILT2 antibodies has re-established the cytotoxic activity of NK cells in vivo [65]. Since PD1/PDL1 and HLA-G/ILT2 appear to function as independent mechanisms, the combination of both therapies has been shown to restore antitumor activity in a variety of tumor cells [66].

Our results have established the basis for future studies investigating the functional relationship between HLA-G variants and HLA-G expression levels in HNSCC patients. We plan to analyse other HLA-G 3′UTR variants and determine their impact on HLA-G expression in HNSCC patients, as has been shown in other tumor types [53,67,68]. HLA-G variants seem to be promising biomarkers for the prediction of disease outcomes and therapy responses in HNSCC patients.

## 5. Conclusions

This study evaluated the association between the HLA-G 14 bp ins/del polymorphism, HLA-G, ILT2 and ILT4 mRNA expression and clinical variables (age at onset, TNM staging system and p16 positivity) in HNSCC patients. Our results revealed an association between HLA-G mRNA expression and tumors in the T4 stage in non-metastatic HNSCC patients. Furthermore, a significantly higher ILT4 mRNA expression was noted in tumors in the T1+T2 stage compared to those in the T3 stage. Thus, increased HLA-G and ILTs expression might serve as potential biomarkers in the diagnosis and treatment of patients with HNSCC.

## Figures and Tables

**Table 1:** Demographic and clinical characteristics of head and neck squamous cell carcinoma (HNSCC) patients and controls.

Parameter	HNSCC Patients (n = 34)	Controls (n = 98)	*p*-Value
Males/Females	33/1	92/6	0.79
Age at examination (mean ± SD, years)	62.32 ± 6.63	63.10 ± 7.41	0.57
Age at onset (mean ± SD, years)	61.65 ± 6.18	-	-
Primary diagnosis	31	-	-
Relapse	3	-	-
Tobacco consumption (yes/no)	30/4	-	-
Tumor staging			
T1	1	-	-
T2	6	-	-
T3	6	-	-
T4	16	-	-
Not available	5		
Lymph nodes			
N0	13	-	-
N1	6	-	-
N2	10	-	-
N3	5	-	-
Metastasis		-	
M0	28	-	-
M1	6	-	-
p16 positivity			
yes	6	-	-
no	13	-	-
Not available	15	-	-

Data are shown as the mean with standard deviation. SD: standard deviation; n: number. Age differences between the two groups were examined using Student’s *t* test with Welch’s correction. Gender differences were assessed using χ^2^ test.

**Table 2:** HLA-G 14 bp ins/del allele and genotype frequencies in HNSCC patients and controls.

SNP/ Model	Allele/ Genotype	HNSCC Patients (n = 34)	Controls	Logistic Regression Analysis	
			(n = 98)	*p*-Value	OR (95% CI)
14 bp ins/del	14 bp del	36 (52.94%)	112 (57.14%)		
	14 bp ins	32 (47.06%)	84 (42.86%)	0.65	1.19 (0.68–2.06)
	14 bp del/del	7 (20.59%)	31 (31.63%)		1.00
Codominant	14 bp ins/del	22 (64.70%)	50 (51.02%)	0.38	1.87 (0.71–4.94)
	14 bp ins/ins	5 (14.71%)	17 (17.35%)		1.19 (0.32–4.39)
	14 bp del/del	7 (20.59%)	31 (31.63%)		1.00
Dominant	14 bp ins/del + 14 bp ins/ins	27 (79.41%)	67 (68.37%)	0.26	1.70 (0.66–4.37)
	14 bp del/del + 14 bp ins/del	29 (85.29%)	81 (82.65%)		1.00
Recessive	14 bp ins/ins	5 (14.71%)	17 (17.35%)	0.62	0.76 (0.25–2.27)

The frequencies of alleles and genotypes are presented in parentheses. *p*, OR and 95% CI values for genotype comparisons were adjusted for age and sex. Del: deletion; ins: insertion; n: number; CI: confidence interval; OR: odds ratio. *p* values less than 0.05 were considered statistically significant.

**Table 3:** Impact of HLA-G 14 bp ins/del polymorphism on HLA-G mRNA expression in HNSCC patients.

HLA-G Genotype	Relative HLA-G Expression (Mean ± SD)	*p*-Value
14 bp del/del (n = 7)	1.483 ± 1.931	0.4794 (del vs. H)
14 bp ins/del (n = 22)	0.9012 ± 1.271	0.0641 (H vs. ins)
14 bp ins/ins (n = 5)	0.3408 ± 0.2228	0.1721 (del vs. ins)

*p* value assessed using Student’s *t* test with Welch’s correction. n: number; SD: standard deviation; del: deletion; ins: insertion; H: heterozygous genotype. *p* values less than 0.05 were considered statistically significant.

**Table 4:** Association of HLA-G mRNA expression with clinical variables in HNSCC patients.

Clinical Variables	Relative HLA-G Expression (Mean ± SD)	*p*-Value
**Age at onset**		
I: 49 to 60 (n = 13)	0.8367 ± 1.591	0.5901 (I vs. II)
II: 61 to 66 (n = 16)	1.139 ± 1.338	0.2129 (II vs. III)
III: 67 to 81 (n = 5)	0.5612 ± 0.6491	0.6096 (I vs. III)
**Tumor staging**		
I. T1 + T2 (n = 7)	0.6519 ± 1.055	0.8163 (I vs. II)
II. T3 (n = 6)	0.5507 ± 0.3180	0.0601 (II vs. III)
III. T4 (n = 16)	1.454 ± 1.717	0.1878 (I vs. III)
**Lymph nodes**		
I. N0 (n = 13)	1.044 ± 1.204	0.0754 (I vs. II)
II. N1 (n = 6)	0.3655 ± 0.2839	0.1373 (II vs. III)
III. N2 + N3 (n = 15)	1.076 ± 1.695	0.9547 (I vs. III)
**Metastasis**		
M0 (n = 28)	0.9180 ± 1.393	
M1 (n = 6)	1.035 ± 1.261	0.8460
**p16 positivity**		
Yes (n = 6)	0.3532 ± 0.2838	
No (n = 13)	1.009 ± 1.442	0.1392

*p* value assessed using Student’s *t* test with Welch’s correction. n: number; SD: standard deviation. *p* values less than 0.05 were considered statistically significant.

**Table 5:** Association of HLA-G mRNA expression with clinical variables in non-metastatic HNSCC patients.

Clinical Variables	Relative HLA-G Expression (Mean ± SD)	*p*-Value
**Age at onset**		
I: 49 to 60 (n = 11)	0.7775 ± 1.663	0.4538 (I vs. II)
II: 61 to 66 (n = 14)	1.255 ± 1.392	0.2598 (II vs. III)
III: 67 to 81 (n = 4)	0.6343 ± 0.7254	0.8209 (I vs. III)
**Tumor staging**		
I. T1 + T2 (n = 6)	0.2680 ± 0.3137	0.2192 (I vs. II)
II. T3 (n = 5)	0.5396 ± 0.3542	0.0832 (II vs. III)
III. T4 (n = 14)	1.480 ± 1.801	**0.0289** (I vs. III)
**Lymph nodes**		
I. N0 (n = 11)	0.9721 ± 1.250	0.1522 (I vs. II)
II. N1 (n = 6)	0.3655 ± 0.2839	0.1884 (II vs. III)
III. N2 + N3 (n = 11)	1.165 ± 1.839	0.7768 (I vs. III)
**p16 positivity**		
Yes (n = 5)	0.4134 ± 0.2710	
No (n = 11)	0.9813 ± 1.497	0.2498

*p* value assessed using Student’s *t* test with Welch’s correction. n: number; SD: standard deviation. *p* values less than 0.05 were considered statistically significant.

**Table 6:** Association of ILT2 and ILT4 mRNA expression with clinical variables in HNSCC patients.

Clinical Variables	Relative ILT2 Expression (Mean ± SD)	*p*-Value	Relative ILT4 Expression (Mean ± SD)	*p*-Value
**Age at onset**				
I: 49 to 60 (n = 13)	0.2474 ± 0.4098	0.2507 (I vs. II)	0.1152 ± 0.1267	0.7403 (I vs. II)
II: 61 to 66 (n = 16)	0.1076 ± 0.0987	0.3834 (II vs. III)	0.1006 ± 0.1027	0.8031 (II vs. III)
III: 67 to 81 (n = 5)	0.2590 ± 0.3418	0.9529 (I vs. III)	0.1156 ± 0.1149	0.9954 (I vs. III)
**Tumor staging**				
I. T1 + T2 (n = 7)	0.2573 ± 0.3015	0.3516 (I vs. II)	0.1880 ± 0.1259	**0.0495** (I vs. II)
II. T3 (n = 6)	0.1320 ± 0.1356	0.7021 (II vs. III)	0.0693 ± 0.0379	0.6005 (II vs. III)
III. T4 (n = 16)	0.1734 ± 0.3641	0.5749 (I vs. III)	0.0868 ± 0.1152	0.0987 (I vs. III)
**Lymph nodes**				
I. N0 (n = 13)	0.1122 ± 0.1306	0.3901 (I vs. II)	0.06531 ± 0.04475	0.1859 (I vs. II)
II. N1 (n = 6)	0.2370 ± 0.3126	0.9341 (II vs. III)	0.1487 ± 0.1297	0.7714 (II vs. III)
III.N2 + N3 (n = 15)	0.2234 ± 0.3781	0.3007 (I vs. III)	0.1297 ± 0.1354	0.1008 (I vs. III)
**Metastasis**				
M0 (n = 28)	0.2107 ± 0.3135		0.1096 ± 0.1079	
M1 (n = 6)	0.05533 ± 0.07611	**0.0269**	0.1028 ± 0.1346	0.9118
**p16 positivity**				
Yes (n = 6)	0.2705 ± 0.1854		0.1227 ± 0.09438	
No (n = 13)	0.1250 ± 0.2257	0.1666	0.09562 ± 0.08947	0.5695

*p* value assessed using Student’s *t* test with Welch’s correction. n: number; SD: standard deviation. *p* values less than 0.05 were considered statistically significant.

## Data Availability

The presented data are available on request from the corresponding author.
